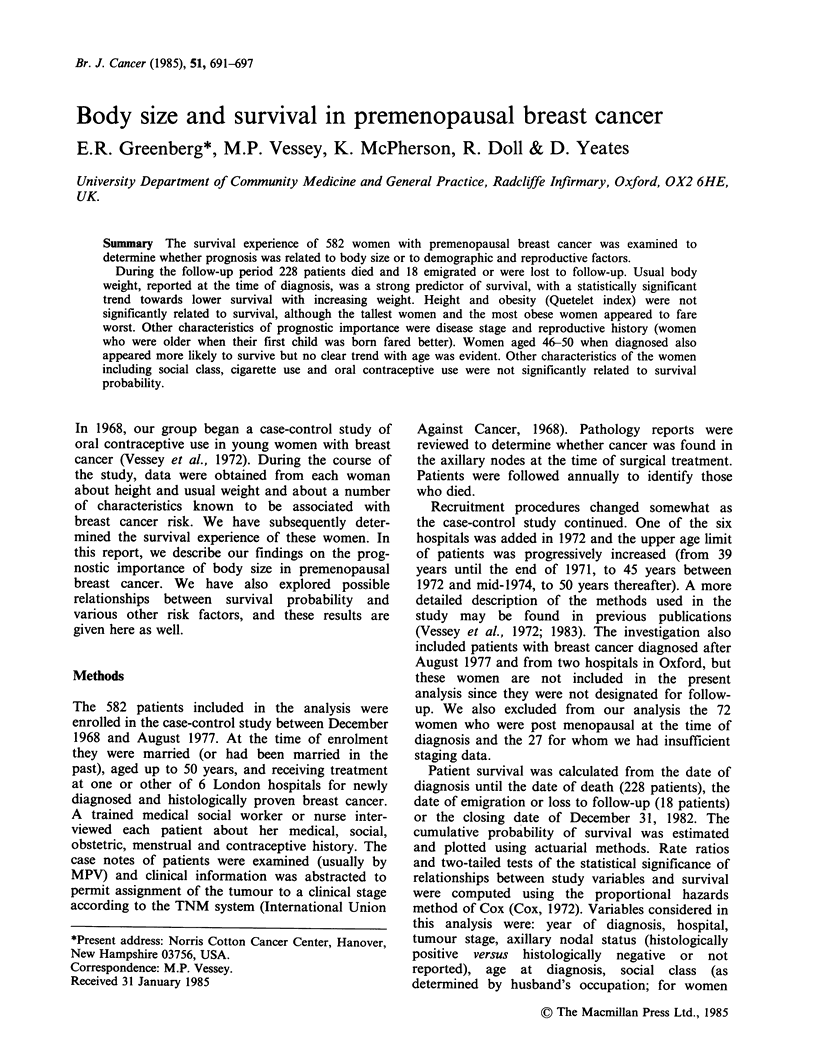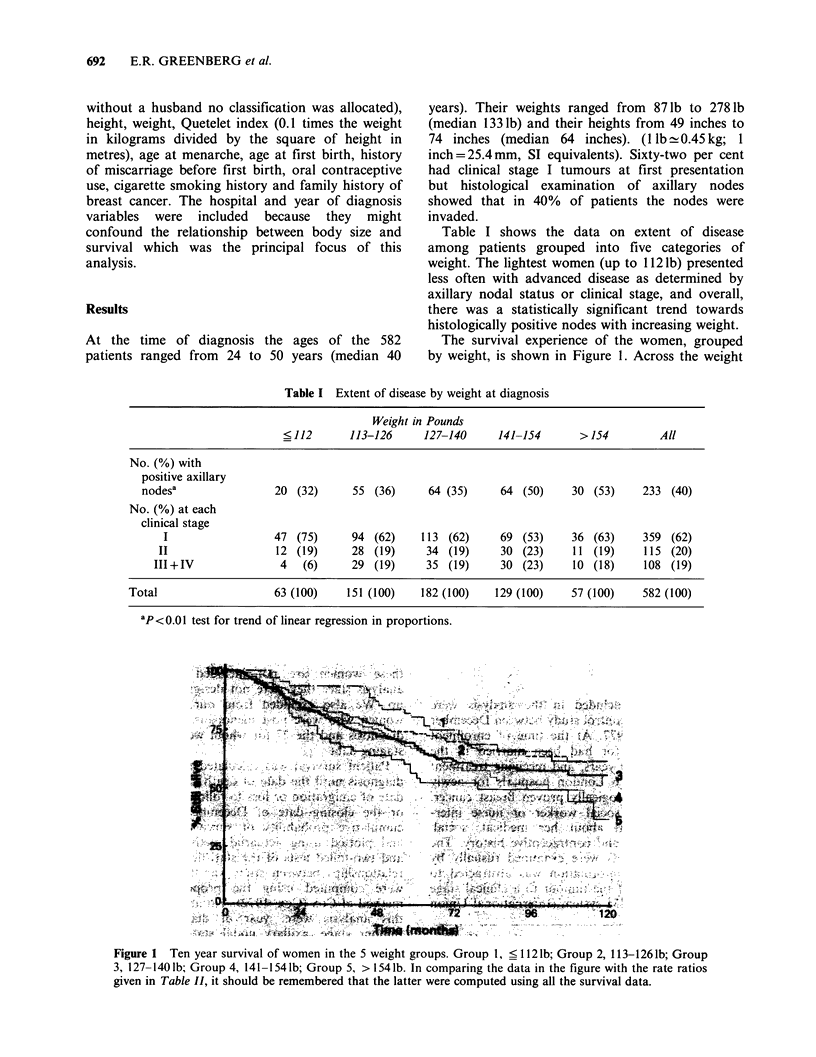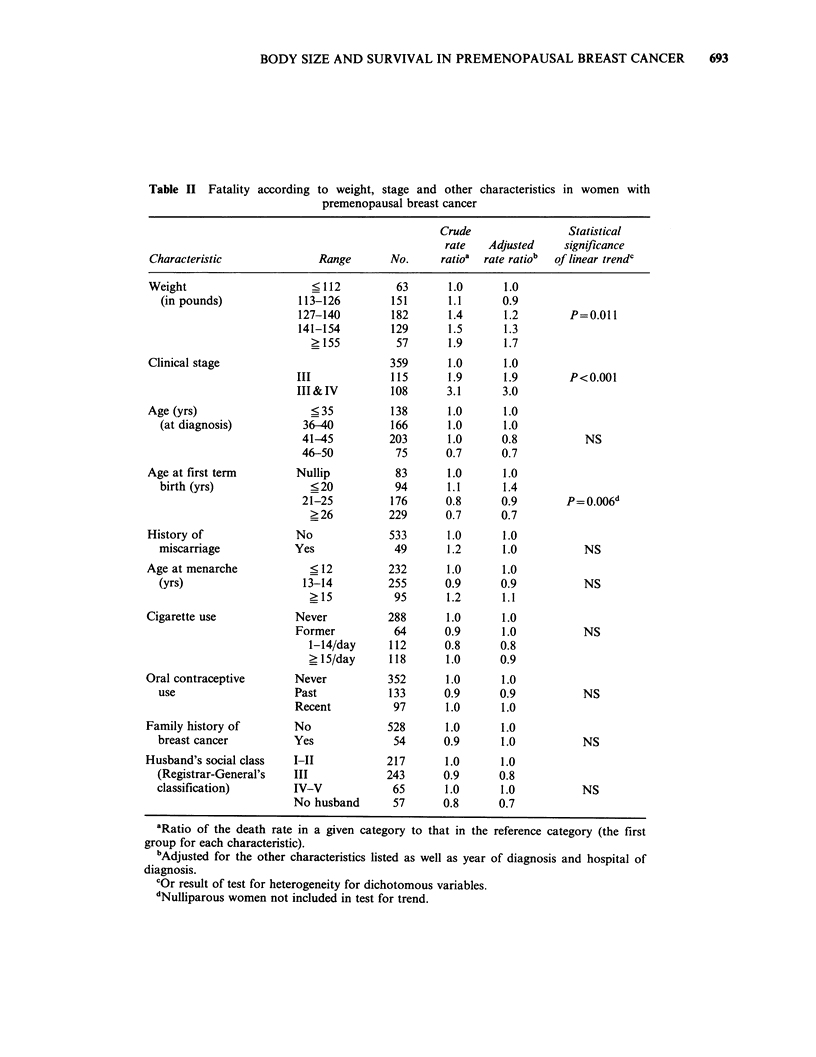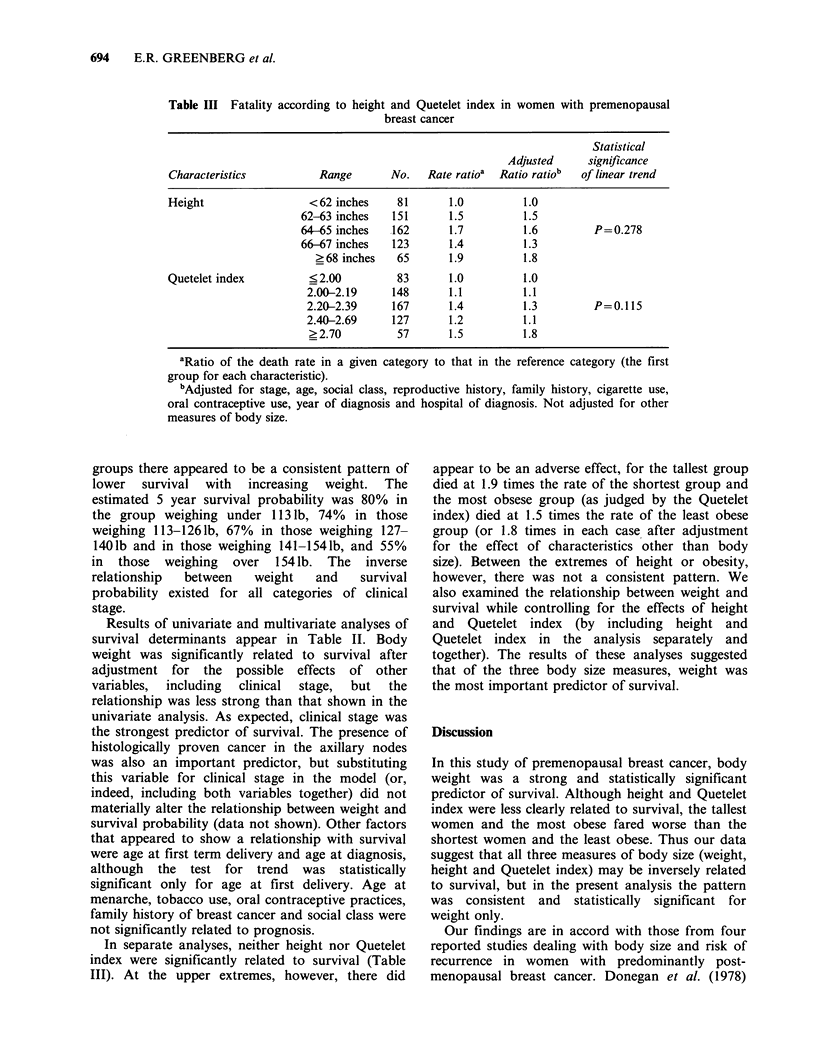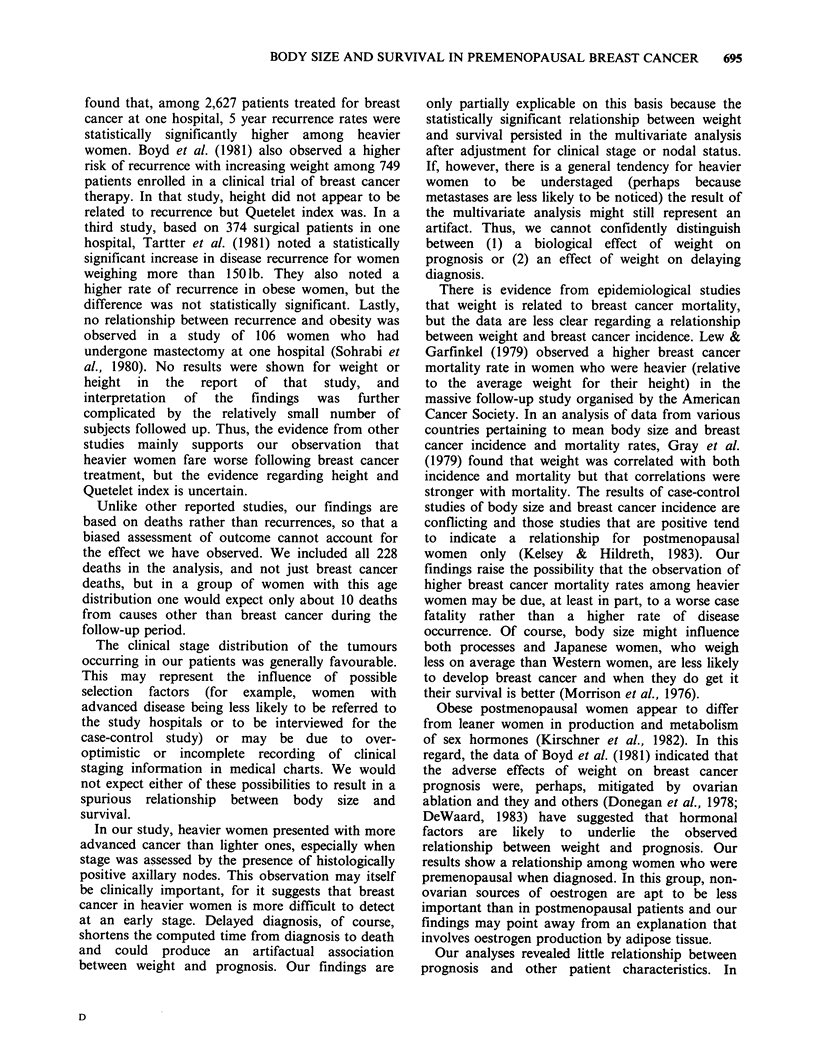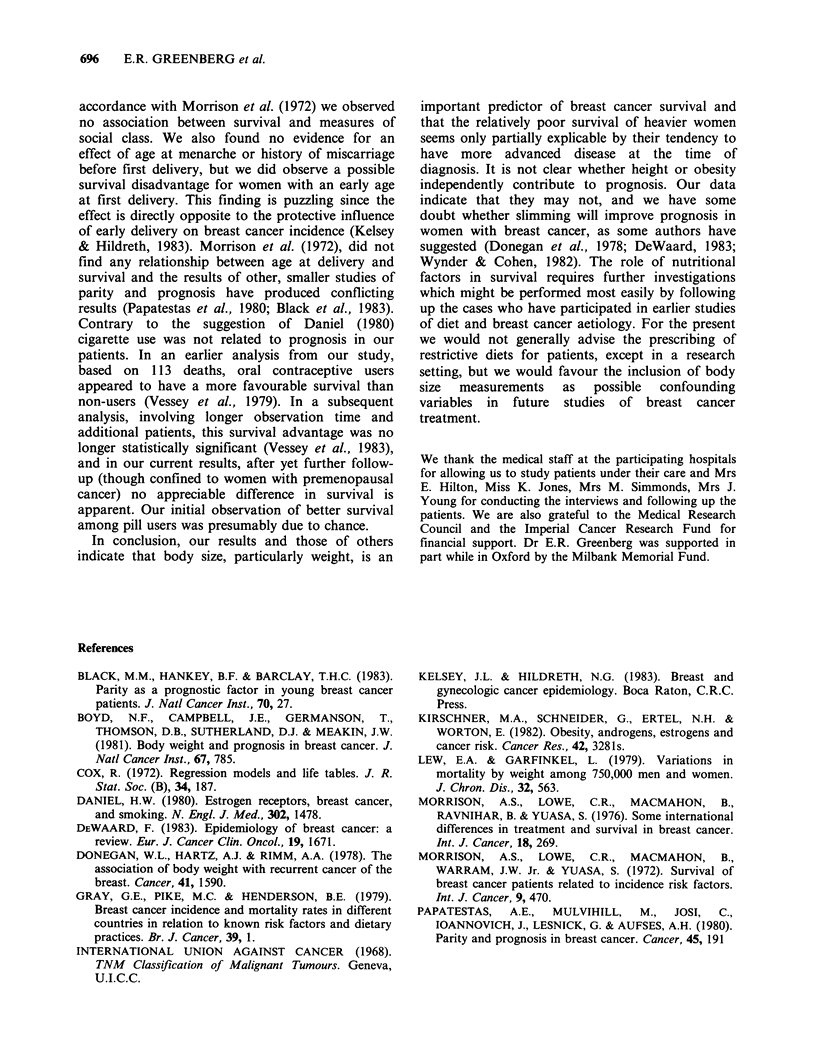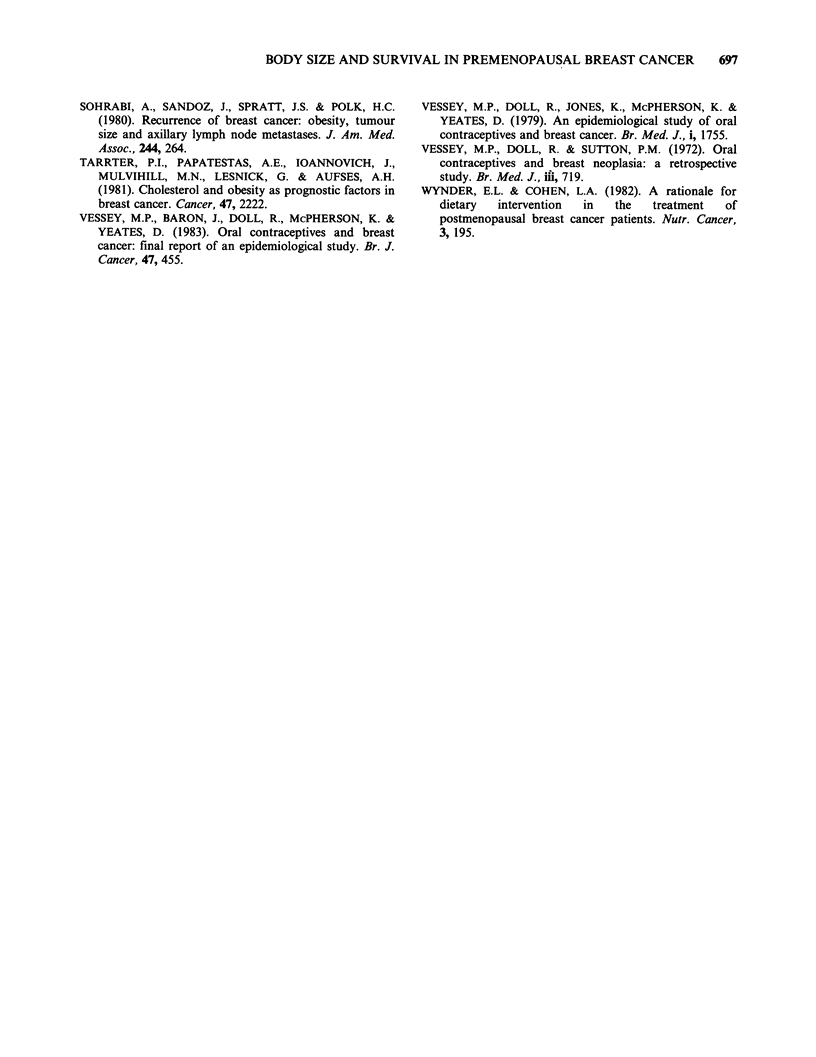# Body size and survival in premenopausal breast cancer.

**DOI:** 10.1038/bjc.1985.104

**Published:** 1985-05

**Authors:** E. R. Greenberg, M. P. Vessey, K. McPherson, R. Doll, D. Yeates

## Abstract

The survival experience of 582 women with premenopausal breast cancer was examined to determine whether prognosis was related to body size or to demographic and reproductive factors. During the follow-up period 228 patients died and 18 emigrated or were lost to follow-up. Usual body weight, reported at the time of diagnosis, was a strong predictor of survival, with a statistically significant trend towards lower survival with increasing weight. Height and obesity (Quetelet index) were not significantly related to survival, although the tallest women and the most obese women appeared to fare worst. Other characteristics of prognostic importance were disease stage and reproductive history (women who were older when their first child was born fared better). Women aged 46-50 when diagnosed also appeared more likely to survive but no clear trend with age was evident. Other characteristics of the women including social class, cigarette use and oral contraceptive use were not significantly related to survival probability.


*Br. J. Cancer* (1985), **51**, 691–697

### Body size and survival in premenopausal breast cancer

E.R. Greenberg*, M.P. Vessey, K. McPherson, R. Doll & D. Yeates

*University Department of Community Medicine and General Practice, Radcliffe Infirmary, Oxford, OX2 6HE, UK.*

**Summary** The survival experience of 582 women with premenopausal breast cancer was examined to determine whether prognosis was related to body size or to demographic and reproductive factors.

During the follow-up period 228 patients died and 18 emigrated or were lost to follow-up. Usual body weight, reported at the time of diagnosis, was a strong predictor of survival, with a statistically significant trend towards lower survival with increasing weight. Height and obesity (Quetelet index) were not significantly related to survival, although the tallest women and the most obese women appeared to fare worst. Other characteristics of prognostic importance were disease stage and reproductive history (women who were older when their first child was born fared better). Women aged 46–50 when diagnosed also appeared more likely to survive but no clear trend with age was evident. Other characteristics of the women including social class, cigarette use and oral contraceptive use were not significantly related to survival probability.

In 1968, our group began a case-control study of oral contraceptive use in young women with breast cancer (Vessey *et al.*, 1972). During the course of the study, data were obtained from each woman about height and usual weight and about a number of characteristics known to be associated with breast cancer risk. We have subsequently determined the survival experience of these women. In this report, we describe our findings on the prognostic importance of body size in premenopausal breast cancer. We have also explored possible relationships between survival probability and various other risk factors, and these results are given here as well.

### Methods

The 582 patients included in the analysis were enrolled in the case-control study between December 1968 and August 1977. At the time of enrolment they were married (or had been married in the past), aged up to 50 years, and receiving treatment at one or other of 6 London hospitals for newly diagnosed and histologically proven breast cancer. A trained medical social worker or nurse interviewed each patient about her medical, social, obstetric, menstrual and contraceptive history. The case notes of patients were examined (usually by MPV) and clinical information was abstracted to permit assignment of the tumour to a clinical stage according to the TNM system (International Union Against Cancer, 1968). Pathology reports were reviewed to determine whether cancer was found in the axillary nodes at the time of surgical treatment. Patients were followed annually to identify those who died.

Recruitment procedures changed somewhat as the case-control study continued. One of the six hospitals was added in 1972 and the upper age limit of patients was progressively increased (from 39 years until the end of 1971, to 45 years between 1972 and mid-1974, to 50 years thereafter). A more detailed description of the methods used in the study may be found in previous publications (Vessey *et al.*, 1972; 1983). The investigation also included patients with breast cancer diagnosed after August 1977 and from two hospitals in Oxford, but these women are not included in the present analysis since they were not designated for follow-up. We also excluded from our analysis the 72 women who were post menopausal at the time of diagnosis and the 27 for whom we had insufficient staging data.

Patient survival was calculated from the date of diagnosis until the date of death (228 patients), the date of emigration or loss to follow-up (18 patients) or the closing date of December 31, 1982. The cumulative probability of survival was estimated and plotted using actuarial methods. Rate ratios and two-tailed tests of the statistical significance of relationships between study variables and survival were computed using the proportional hazards method of Cox (Cox, 1972). Variables considered in this analysis were: year of diagnosis, hospital, tumour stage, axillary nodal status (histologically positive *versus* histologically negative or not reported), age at diagnosis, social class (as determined by husband's occupation; for women

*Present address: Norris Cotton Cancer Center, Hanover, New Hampshire 03756, USA.
Correspondence: M.P. Vessey.
Received 31 January 1985

© The Macmillan Press Ltd., 1985

without a husband no classification was allocated), height, weight, Quetelet index (0.1 times the weight in kilograms divided by the square of height in metres), age at menarche, age at first birth, history of miscarriage before first birth, oral contraceptive use, cigarette smoking history and family history of breast cancer. The hospital and year of diagnosis variables were included because they might confound the relationship between body size and survival which was the principal focus of this analysis.

### Results

At the time of diagnosis the ages of the 582 patients ranged from 24 to 50 years (median 40 years). Their weights ranged from 87 lb to 278 lb (median 133 lb) and their heights from 49 inches to 74 inches (median 64 inches). (1 lb $\simeq$ 0.45 kg; 1 inch = 25.4 mm, SI equivalents). Sixty-two per cent had clinical stage I tumours at first presentation but histological examination of axillary nodes showed that in 40% of patients the nodes were invaded.

Table I shows the data on extent of disease among patients grouped into five categories of weight. The lightest women (up to 112 lb) presented less often with advanced disease as determined by axillary nodal status or clinical stage, and overall, there was a statistically significant trend towards histologically positive nodes with increasing weight.

The survival experience of the women, grouped by weight, is shown in Figure 1. Across the weight

**Table I** Extent of disease by weight at diagnosis

| | *Weight in Pounds* | | | | | |
|---|---|---|---|---|---|---|
| | *≦112* | *113–126* | *127–140* | *141–154* | *>154* | *All* |
| No. (%) with positive axillary nodes[a] | 20 (32) | 55 (36) | 64 (35) | 64 (50) | 30 (53) | 233 (40) |
| No. (%) at each clinical stage | | | | | | |
| I | 47 (75) | 94 (62) | 113 (62) | 69 (53) | 36 (63) | 359 (62) |
| II | 12 (19) | 28 (19) | 34 (19) | 30 (23) | 11 (19) | 115 (20) |
| III+IV | 4 (6) | 29 (19) | 35 (19) | 30 (23) | 10 (18) | 108 (19) |
| Total | 63 (100) | 151 (100) | 182 (100) | 129 (100) | 57 (100) | 582 (100) |

[a]$P < 0.01$ test for trend of linear regression in proportions.

**Figure 1** Ten year survival of women in the 5 weight groups. Group 1, ≦112 lb; Group 2, 113–126 lb; Group 3, 127–140 lb; Group 4, 141–154 lb; Group 5, >154 lb. In comparing the data in the figure with the rate ratios given in *Table II*, it should be remembered that the latter were computed using all the survival data.

**Table II** Fatality according to weight, stage and other characteristics in women with premenopausal breast cancer

| *Characteristic* | *Range* | *No.* | *Crude rate ratio*[a] | *Adjusted rate ratio*[b] | *Statistical significance of linear trend*[c] |
|---|---|---|---|---|---|
| Weight (in pounds) | ≦112 | 63 | 1.0 | 1.0 | |
| | 113–126 | 151 | 1.1 | 0.9 | |
| | 127–140 | 182 | 1.4 | 1.2 | $P=0.011$ |
| | 141–154 | 129 | 1.5 | 1.3 | |
| | ≧155 | 57 | 1.9 | 1.7 | |
| Clinical stage | | 359 | 1.0 | 1.0 | |
| | III | 115 | 1.9 | 1.9 | $P<0.001$ |
| | III & IV | 108 | 3.1 | 3.0 | |
| Age (yrs) (at diagnosis) | ≦35 | 138 | 1.0 | 1.0 | |
| | 36–40 | 166 | 1.0 | 1.0 | |
| | 41–45 | 203 | 1.0 | 0.8 | NS |
| | 46–50 | 75 | 0.7 | 0.7 | |
| Age at first term birth (yrs) | Nullip | 83 | 1.0 | 1.0 | |
| | ≦20 | 94 | 1.1 | 1.4 | |
| | 21–25 | 176 | 0.8 | 0.9 | $P=0.006$[d] |
| | ≧26 | 229 | 0.7 | 0.7 | |
| History of miscarriage | No | 533 | 1.0 | 1.0 | |
| | Yes | 49 | 1.2 | 1.0 | NS |
| Age at menarche (yrs) | ≦12 | 232 | 1.0 | 1.0 | |
| | 13–14 | 255 | 0.9 | 0.9 | NS |
| | ≧15 | 95 | 1.2 | 1.1 | |
| Cigarette use | Never | 288 | 1.0 | 1.0 | |
| | Former | 64 | 0.9 | 1.0 | NS |
| | 1–14/day | 112 | 0.8 | 0.8 | |
| | ≧15/day | 118 | 1.0 | 0.9 | |
| Oral contraceptive use | Never | 352 | 1.0 | 1.0 | |
| | Past | 133 | 0.9 | 0.9 | NS |
| | Recent | 97 | 1.0 | 1.0 | |
| Family history of breast cancer | No | 528 | 1.0 | 1.0 | |
| | Yes | 54 | 0.9 | 1.0 | NS |
| Husband's social class (Registrar-General's classification) | I–II | 217 | 1.0 | 1.0 | |
| | III | 243 | 0.9 | 0.8 | |
| | IV–V | 65 | 1.0 | 1.0 | NS |
| | No husband | 57 | 0.8 | 0.7 | |

[a]Ratio of the death rate in a given category to that in the reference category (the first group for each characteristic).

[b]Adjusted for the other characteristics listed as well as year of diagnosis and hospital of diagnosis.

[c]Or result of test for heterogeneity for dichotomous variables.

[d]Nulliparous women not included in test for trend.

**Table III** Fatality according to height and Quetelet index in women with premenopausal breast cancer

| *Characteristics* | *Range* | *No.* | *Rate ratio*[a] | *Adjusted Ratio ratio*[b] | *Statistical significance of linear trend* |
|---|---|---|---|---|---|
| Height | <62 inches | 81 | 1.0 | 1.0 | |
| | 62–63 inches | 151 | 1.5 | 1.5 | |
| | 64–65 inches | 162 | 1.7 | 1.6 | $P=0.278$ |
| | 66–67 inches | 123 | 1.4 | 1.3 | |
| | ≧68 inches | 65 | 1.9 | 1.8 | |
| Quetelet index | ≦2.00 | 83 | 1.0 | 1.0 | |
| | 2.00–2.19 | 148 | 1.1 | 1.1 | |
| | 2.20–2.39 | 167 | 1.4 | 1.3 | $P=0.115$ |
| | 2.40–2.69 | 127 | 1.2 | 1.1 | |
| | ≧2.70 | 57 | 1.5 | 1.8 | |

[a]Ratio of the death rate in a given category to that in the reference category (the first group for each characteristic).

[b]Adjusted for stage, age, social class, reproductive history, family history, cigarette use, oral contraceptive use, year of diagnosis and hospital of diagnosis. Not adjusted for other measures of body size.

groups there appeared to be a consistent pattern of lower survival with increasing weight. The estimated 5 year survival probability was 80% in the group weighing under 113 lb, 74% in those weighing 113–126 lb, 67% in those weighing 127–140 lb and in those weighing 141–154 lb, and 55% in those weighing over 154 lb. The inverse relationship between weight and survival probability existed for all categories of clinical stage.

Results of univariate and multivariate analyses of survival determinants appear in Table II. Body weight was significantly related to survival after adjustment for the possible effects of other variables, including clinical stage, but the relationship was less strong than that shown in the univariate analysis. As expected, clinical stage was the strongest predictor of survival. The presence of histologically proven cancer in the axillary nodes was also an important predictor, but substituting this variable for clinical stage in the model (or, indeed, including both variables together) did not materially alter the relationship between weight and survival probability (data not shown). Other factors that appeared to show a relationship with survival were age at first term delivery and age at diagnosis, although the test for trend was statistically significant only for age at first delivery. Age at menarche, tobacco use, oral contraceptive practices, family history of breast cancer and social class were not significantly related to prognosis.

In separate analyses, neither height nor Quetelet index were significantly related to survival (Table III). At the upper extremes, however, there did appear to be an adverse effect, for the tallest group died at 1.9 times the rate of the shortest group and the most obsese group (as judged by the Quetelet index) died at 1.5 times the rate of the least obese group (or 1.8 times in each case after adjustment for the effect of characteristics other than body size). Between the extremes of height or obesity, however, there was not a consistent pattern. We also examined the relationship between weight and survival while controlling for the effects of height and Quetelet index (by including height and Quetelet index in the analysis separately and together). The results of these analyses suggested that of the three body size measures, weight was the most important predictor of survival.

### Discussion

In this study of premenopausal breast cancer, body weight was a strong and statistically significant predictor of survival. Although height and Quetelet index were less clearly related to survival, the tallest women and the most obese fared worse than the shortest women and the least obese. Thus our data suggest that all three measures of body size (weight, height and Quetelet index) may be inversely related to survival, but in the present analysis the pattern was consistent and statistically significant for weight only.

Our findings are in accord with those from four reported studies dealing with body size and risk of recurrence in women with predominantly post-menopausal breast cancer. Donegan *et al.* (1978)

found that, among 2,627 patients treated for breast cancer at one hospital, 5 year recurrence rates were statistically significantly higher among heavier women. Boyd *et al.* (1981) also observed a higher risk of recurrence with increasing weight among 749 patients enrolled in a clinical trial of breast cancer therapy. In that study, height did not appear to be related to recurrence but Quetelet index was. In a third study, based on 374 surgical patients in one hospital, Tartter *et al.* (1981) noted a statistically significant increase in disease recurrence for women weighing more than 150lb. They also noted a higher rate of recurrence in obese women, but the difference was not statistically significant. Lastly, no relationship between recurrence and obesity was observed in a study of 106 women who had undergone mastectomy at one hospital (Sohrabi *et al.*, 1980). No results were shown for weight or height in the report of that study, and interpretation of the findings was further complicated by the relatively small number of subjects followed up. Thus, the evidence from other studies mainly supports our observation that heavier women fare worse following breast cancer treatment, but the evidence regarding height and Quetelet index is uncertain.

Unlike other reported studies, our findings are based on deaths rather than recurrences, so that a biased assessment of outcome cannot account for the effect we have observed. We included all 228 deaths in the analysis, and not just breast cancer deaths, but in a group of women with this age distribution one would expect only about 10 deaths from causes other than breast cancer during the follow-up period.

The clinical stage distribution of the tumours occurring in our patients was generally favourable. This may represent the influence of possible selection factors (for example, women with advanced disease being less likely to be referred to the study hospitals or to be interviewed for the case-control study) or may be due to over-optimistic or incomplete recording of clinical staging information in medical charts. We would not expect either of these possibilities to result in a spurious relationship between body size and survival.

In our study, heavier women presented with more advanced cancer than lighter ones, especially when stage was assessed by the presence of histologically positive axillary nodes. This observation may itself be clinically important, for it suggests that breast cancer in heavier women is more difficult to detect at an early stage. Delayed diagnosis, of course, shortens the computed time from diagnosis to death and could produce an artifactual association between weight and prognosis. Our findings are only partially explicable on this basis because the statistically significant relationship between weight and survival persisted in the multivariate analysis after adjustment for clinical stage or nodal status. If, however, there is a general tendency for heavier women to be understaged (perhaps because metastases are less likely to be noticed) the result of the multivariate analysis might still represent an artifact. Thus, we cannot confidently distinguish between (1) a biological effect of weight on prognosis or (2) an effect of weight on delaying diagnosis.

There is evidence from epidemiological studies that weight is related to breast cancer mortality, but the data are less clear regarding a relationship between weight and breast cancer incidence. Lew & Garfinkel (1979) observed a higher breast cancer mortality rate in women who were heavier (relative to the average weight for their height) in the massive follow-up study organised by the American Cancer Society. In an analysis of data from various countries pertaining to mean body size and breast cancer incidence and mortality rates, Gray *et al.* (1979) found that weight was correlated with both incidence and mortality but that correlations were stronger with mortality. The results of case-control studies of body size and breast cancer incidence are conflicting and those studies that are positive tend to indicate a relationship for postmenopausal women only (Kelsey & Hildreth, 1983). Our findings raise the possibility that the observation of higher breast cancer mortality rates among heavier women may be due, at least in part, to a worse case fatality rather than a higher rate of disease occurrence. Of course, body size might influence both processes and Japanese women, who weigh less on average than Western women, are less likely to develop breast cancer and when they do get it their survival is better (Morrison *et al.*, 1976).

Obese postmenopausal women appear to differ from leaner women in production and metabolism of sex hormones (Kirschner *et al.*, 1982). In this regard, the data of Boyd *et al.* (1981) indicated that the adverse effects of weight on breast cancer prognosis were, perhaps, mitigated by ovarian ablation and they and others (Donegan *et al.*, 1978; DeWaard, 1983) have suggested that hormonal factors are likely to underlie the observed relationship between weight and prognosis. Our results show a relationship among women who were premenopausal when diagnosed. In this group, non-ovarian sources of oestrogen are apt to be less important than in postmenopausal patients and our findings may point away from an explanation that involves oestrogen production by adipose tissue.

Our analyses revealed little relationship between prognosis and other patient characteristics. In accordance with Morrison *et al.* (1972) we observed no association between survival and measures of social class. We also found no evidence for an effect of age at menarche or history of miscarriage before first delivery, but we did observe a possible survival disadvantage for women with an early age at first delivery. This finding is puzzling since the effect is directly opposite to the protective influence of early delivery on breast cancer incidence (Kelsey & Hildreth, 1983). Morrison *et al.* (1972), did not find any relationship between age at delivery and survival and the results of other, smaller studies of parity and prognosis have produced conflicting results (Papatestas *et al.*, 1980; Black *et al.*, 1983). Contrary to the suggestion of Daniel (1980) cigarette use was not related to prognosis in our patients. In an earlier analysis from our study, based on 113 deaths, oral contraceptive users appeared to have a more favourable survival than non-users (Vessey *et al.*, 1979). In a subsequent analysis, involving longer observation time and additional patients, this survival advantage was no longer statistically significant (Vessey *et al.*, 1983), and in our current results, after yet further follow-up (though confined to women with premenopausal cancer) no appreciable difference in survival is apparent. Our initial observation of better survival among pill users was presumably due to chance.

In conclusion, our results and those of others indicate that body size, particularly weight, is an important predictor of breast cancer survival and that the relatively poor survival of heavier women seems only partially explicable by their tendency to have more advanced disease at the time of diagnosis. It is not clear whether height or obesity independently contribute to prognosis. Our data indicate that they may not, and we have some doubt whether slimming will improve prognosis in women with breast cancer, as some authors have suggested (Donegan *et al.*, 1978; DeWaard, 1983; Wynder & Cohen, 1982). The role of nutritional factors in survival requires further investigations which might be performed most easily by following up the cases who have participated in earlier studies of diet and breast cancer aetiology. For the present we would not generally advise the prescribing of restrictive diets for patients, except in a research setting, but we would favour the inclusion of body size measurements as possible confounding variables in future studies of breast cancer treatment.

We thank the medical staff at the participating hospitals for allowing us to study patients under their care and Mrs E. Hilton, Miss K. Jones, Mrs M. Simmonds, Mrs J. Young for conducting the interviews and following up the patients. We are also grateful to the Medical Research Council and the Imperial Cancer Research Fund for financial support. Dr E.R. Greenberg was supported in part while in Oxford by the Milbank Memorial Fund.

### References

BLACK, M.M., HANKEY, B.F. & BARCLAY, T.H.C. (1983). Parity as a prognostic factor in young breast cancer patients. *J. Natl Cancer Inst.*, **70**, 27.

BOYD, N.F., CAMPBELL, J.E., GERMANSON, T., THOMSON, D.B., SUTHERLAND, D.J. & MEAKIN, J.W. (1981). Body weight and prognosis in breast cancer. *J. Natl Cancer Inst.*, **67**, 785.

COX, R. (1972). Regression models and life tables. *J. R. Stat. Soc.* (B), **34**, 187.

DANIEL, H.W. (1980). Estrogen receptors, breast cancer, and smoking. *N. Engl. J. Med.*, **302**, 1478.

DEWAARD, F. (1983). Epidemiology of breast cancer: a review. *Eur. J. Cancer Clin. Oncol.*, **19**, 1671.

DONEGAN, W.L., HARTZ, A.J. & RIMM, A.A. (1978). The association of body weight with recurrent cancer of the breast. *Cancer*, **41**, 1590.

GRAY, G.E., PIKE, M.C. & HENDERSON, B.E. (1979). Breast cancer incidence and mortality rates in different countries in relation to known risk factors and dietary practices. *Br. J. Cancer*, **39**, 1.

INTERNATIONAL UNION AGAINST CANCER (1968). *TNM Classification of Malignant Tumours*. Geneva, U.I.C.C.

KELSEY, J.L. & HILDRETH, N.G. (1983). Breast and gynecologic cancer epidemiology. Boca Raton, C.R.C. Press.

KIRSCHNER, M.A., SCHNEIDER, G., ERTEL, N.H. & WORTON, E. (1982). Obesity, androgens, estrogens and cancer risk. *Cancer Res.*, **42**, 3281s.

LEW, E.A. & GARFINKEL, L. (1979). Variations in mortality by weight among 750,000 men and women. *J. Chron. Dis.*, **32**, 563.

MORRISON, A.S., LOWE, C.R., MACMAHON, B., RAVNIHAR, B. & YUASA, S. (1976). Some international differences in treatment and survival in breast cancer. *Int. J. Cancer*, **18**, 269.

MORRISON, A.S., LOWE, C.R., MACMAHON, B., WARRAM, J.W. Jr. & YUASA, S. (1972). Survival of breast cancer patients related to incidence risk factors. *Int. J. Cancer*, **9**, 470.

PAPATESTAS, A.E., MULVIHILL, M., JOSI, C., IOANNOVICH, J., LESNICK, G. & AUFSES, A.H. (1980). Parity and prognosis in breast cancer. *Cancer*, **45**, 191

SOHRABI, A., SANDOZ, J., SPRATT, J.S. & POLK, H.C. (1980). Recurrence of breast cancer: obesity, tumour size and axillary lymph node metastases. *J. Am. Med. Assoc.*, **244**, 264.

TARRTER, P.I., PAPATESTAS, A.E., IOANNOVICH, J., MULVIHILL, M.N., LESNICK, G. & AUFSES, A.H. (1981). Cholesterol and obesity as prognostic factors in breast cancer. *Cancer*, **47**, 2222.

VESSEY, M.P., BARON, J., DOLL, R., MCPHERSON, K. & YEATES, D. (1983). Oral contraceptives and breast cancer: final report of an epidemiological study. *Br. J. Cancer*, **47**, 455.

VESSEY, M.P., DOLL, R., JONES, K., MCPHERSON, K. & YEATES, D. (1979). An epidemiological study of oral contraceptives and breast cancer. *Br. Med. J.*, **i**, 1755.

VESSEY, M.P., DOLL, R. & SUTTON, P.M. (1972). Oral contraceptives and breast neoplasia: a retrospective study. *Br. Med. J.*, **iii**, 719.

WYNDER, E.L. & COHEN, L.A. (1982). A rationale for dietary intervention in the treatment of postmenopausal breast cancer patients. *Nutr. Cancer*, **3**, 195.